# USPSTF Colorectal Cancer Screening Recommendation and Uptake for Individuals Aged 45 to 49 Years

**DOI:** 10.1001/jamanetworkopen.2024.36358

**Published:** 2024-10-03

**Authors:** Sunny Siddique, Rong Wang, Faiza Yasin, Jacquelyne J. Gaddy, Lan Zhang, Cary P. Gross, Xiaomei Ma

**Affiliations:** 1 Department of Chronic Disease Epidemiology, Yale School of Public Health, New Haven, Connecticut; 2 Medical Oncology, Department of Internal Medicine, Yale School of Medicine, New Haven, Connecticut; 3 Cancer Outcomes Public Policy and Effectiveness Research (COPPER) Center, Yale Cancer Center, New Haven, Connecticut; 4 General Medicine, Department of Internal Medicine, Yale School of Medicine, New Haven, Connecticut

## Abstract

**Importance:**

In May 2021, the US Preventive Services Task Force (USPSTF) issued a grade B recommendation encouraging colorectal cancer (CRC) screening among average-risk individuals aged 45 to 49 years. The patterns of screening uptake and possible socioeconomic disparities in screening in this age group remain unknown.

**Objective:**

To evaluate changes in CRC screening uptake among average-risk individuals aged 45 to 49 years after the USPSTF recommendation was issued in 2021.

**Design, Setting, and Participants:**

This retrospective cohort study used deidentified claims data from commercially insured Blue Cross Blue Shield beneficiaries aged 45 to 49 years across the US between January 1, 2017, and December 31, 2022.

**Exposure:**

Publication of the May 2021 USPSTF CRC screening recommendation for adults aged 45 to 49 years.

**Main Outcomes and Measures:**

Absolute and relative changes in screening uptake were compared between a 20-month period preceding (May 1, 2018, to December 31, 2019) and a 20-month period following (May 1, 2021, to December, 31, 2022) the USPSTF recommendation. Interrupted time-series analysis and autoregressive integrated moving average models were used to evaluate changes in screening rates, adjusting for temporal autocorrelation and seasonality.

**Results:**

In this cohort study of 10 221 114 distinct beneficiaries aged 45 to 49 years (mean [SD] age, 47.04 [1.41] years; 51.04% female), bimonthly mean (SD) numbers of average-risk beneficiaries were 3 213 935 (31 508) and 2 923 327 (105 716) in the prerecommendation and postrecommendation periods, respectively. Mean (SD) screening uptake increased from 0.50% (0.02%) to 1.51% (0.59%) between the 2 periods (*P* < .001), representing an absolute change of 1.01 percentage points (95% CI, 0.62-1.40 percentage points) but no significant relative change (202.51%; 95% CI, −30.59% to 436.87%). Compared with average-risk beneficiaries residing in areas with the lowest socioeconomic status (SES), those residing in areas with the highest SES experienced the largest absolute change in screening (1.25 [95% CI, 0.77-1.74] percentage points vs 0.75 [95% CI, 0.47-1.02] percentage points), but relative changes were not significant (214.01% [95% CI, −30.91% to 461.15%] vs 167.73% [95% CI, −16.30% to 352.62%]). After the recommendation was issued, the screening uptake rate also increased fastest among average-risk beneficiaries residing in the areas with highest SES (0.24 [95% CI, 0.23-0.25] percentage points every 2 months) and metropolitan areas (0.20 [95% CI, 0.19-0.21] percentage points every 2 months).

**Conclusions and Relevance:**

This study found that among privately insured beneficiaries aged 45 to 49 years, CRC screening uptake increased after the USPSTF recommendation, with potential disparities based on SES and locality.

## Key Points

**Question:**

Was the US Preventive Services Task Force (USPSTF) 2021 recommendation for average-risk individuals aged 45 to 49 years to receive colorectal cancer (CRC) screening associated with changes in screening uptake?

**Findings:**

In this nationwide cohort study of 10 221 114 privately insured individuals aged 45 to 49 years, mean rates of CRC screening uptake increased significantly in the 20 months after the USPSTF recommendation vs the 20 months before. Those residing in areas with high socioeconomic status and metropolitan areas experienced a higher increase.

**Meaning:**

This study found that uptake of CRC screening increased after the USPSTF recommendation, but the increase was uneven based on socioeconomic status and locality.

## Teaser

This cohort study examines whether the 2021 US Preventive Services Task Force (USPSTF) recommendation for colorectal cancer screening among individuals aged 45 to 49 years with average risk was associated with changes in screening uptake.

## Introduction

Measurable decreases in colorectal cancer (CRC) incidence and mortality have been attributed to increased screening.^1,2^ In June 2016, the US Preventive Services Task Force (USPSTF) concluded with high certainty that screening for CRC of average-risk, asymptomatic adults aged 50 to 75 years is of substantial net benefit.^3^ Since then, due to the rising incidence of CRC among younger individuals, CRC screening guidelines have encouraged individuals aged 45 to 49 years to receive screening.^4,5^ In May 2018, the American Cancer Society (ACS) issued a qualified recommendation that adults with average risk who are aged 45 years or older undergo regular CRC screening.^6^ Following this recommendation, CRC screening among individuals aged 45 to 49 years more than doubled according to an ACS study of approximately 5800 individuals included in the 2018 National Health Interview Survey.^7^ Subsequently, in May 2021, the USPSTF issued a grade B recommendation for CRC screening in adults aged 45 to 49 years, mandating insurance coverage for this preventive service.^5,8^ Despite these efforts, screening remains below the national target of 80% among adults aged 45 years or older, with particularly low screening observed among individuals aged 45 to 49 years; those identifying as American Indian or Alaska Native, Asian, or Hispanic; individuals born outside the US; and individuals with income below 100% of the federal poverty level.^4,9^

The association between evolving recommendations and uptake of CRC screening among individuals aged 45 to 49 years is not well understood. Although access to health insurance and a usual source of health care have been identified as key promoters of discussion, recommendation, and delivery of screening, little is known about disparities in screening access in the insured population and the potential contribution of socioeconomic status (SES) in screening disparities in this newly included cohort of individuals.^10^ As observed in other age groups, screening uptake may have been lower than expected among those aged 45 to 49 years due to differences in screening access and uptake by SES.^11,12^ There are also concerns that detection of earlier-stage disease in younger individuals unnecessarily exposes this group of individuals to medical complications such as perforation, bleeding, and risks associated with sedation and leads to overdetection of adenomas.^7,13,14^ In addition, it is predicted that lowering the screening age from 50 to 45 years requires 810 additional colonoscopies per 1000 persons screened using a colonoscopy-based screening strategy, substantially increasing financial costs.^15,16^ Furthermore, different recommendations over time may have led to public uncertainty about CRC screening, potentially reducing the impact of future guideline changes. These public health implications warrant the evaluation of CRC screening uptake among individuals aged 45 to 49 years relative to the recently revised guidelines.

In the current study, we assessed the association between the May 2021 USPSTF recommendation and CRC screening uptake in a large cohort of privately insured individuals aged 45 to 49 years across the US. We conducted one of the first and largest studies to identify possible disparities in screening in this age group as well as recent rates in screening uptake after the recommendation was issued.

## Methods

### Data Source and Cohort Selection

We conducted a retrospective cohort study using deidentified commercial claims data from the Blue Cross Blue Shield (BCBS) Axis. BCBS is the largest provider of commercial insurance in the US, covering approximately one-third of the US population. The BCBS Axis includes administrative claims data from more than 92% of physicians and 96% of hospitals nationwide, and it is the only data resource in the health care industry that includes information from every zip code in the US.^17^ As these data are deidentified, the Yale University institutional review board deemed our study to be non–human participant research, and no informed consent was required. We followed the Strengthening the Reporting of Observational Studies in Epidemiology (STROBE) reporting guideline.

We categorized the 72 months from January 1, 2017, through December 31, 2022, into 36 consecutive 2-month periods to capture potential temporal changes in screening patterns and analyze critical short-term epidemiologic phenomena (eg, several waves of the COVID-19 pandemic) that may have been associated with CRC screening uptake. For each period, the denominator in our calculation of CRC screening uptake included beneficiaries aged 45 to 49 years who had (1) BCBS as the primary insurance for at least 12 months prior to the start of the period and remained enrolled through the end of the 2-month period and (2) did not receive CRC screening or related procedures in the 12 months preceding the 2-month period. Our numerator included the subset of the denominator who received CRC screening (eTable 1 in Supplement 1). Some individuals were screened more than once in the same period. Those who were screened more than once in the same period were counted as only 1 individual in the numerator when we evaluated overall screening uptake (ie, CRC screening [yes or no]). With use of previously published methods, CRC screening was identified using claims for the following procedures: fecal occult blood test, fecal immunochemical test (FIT), stool DNA test, flexible sigmoidoscopy, double-contrast barium enema, colonoscopy, and computed tomography colonography (eTable 2 in Supplement 1).^18,19,20,21^ To distinguish screening-related procedures from diagnostic procedures, only outpatient procedures were included, and individuals with claims for gastrointestinal tract symptoms, including abdominal pain, altered bowel habits, weight loss, iron-deficiency anemia, positive fecal occult blood test result, and gastrointestinal bleeding, within the 3 months preceding the period were excluded (eTable 2 in Supplement 1).^22^ Individuals with a history of CRC were censored before the month of the first claim with a CRC diagnosis code.

### Other Characteristics Evaluated

As the USPSTF 2021 recommendation for starting CRC screening at the age of 45 years applied to individuals at average risk for CRC, we assessed an individual’s CRC risk (eMethods and eTable 2 in Supplement 1). We obtained demographic data of the beneficiaries from BCBS; age and sex were known for all, while imputed data on race and ethnicity were only available for about one-third of beneficiaries. Race and ethnicity information in BCBS is imputed using multilevel modeling among beneficiaries with sufficient identifying information at the individual or household level.^23^ We included race and ethnicity in the analysis to assess potential disparities in screening by these factors; categories were Hispanic, non-Hispanic Asian (hereafter, *Asian*), non-Hispanic Black (hereafter, *Black*), non-Hispanic Native American or Pacific Islander (hereafter, *Native American or Pacific Islander*), non-Hispanic White (hereafter, *White*), and unknown. Additionally, the Social Deprivation Index (SDI), a composite measure of area-level deprivation based on 7 demographic characteristics (proportion living in poverty, with less than 12 years of education, of single-parent households, living in rented housing units, living in overcrowded housing units, of households without a car, and of unemployed adults younger than 65 years) collected in the American Community Survey, was calculated using beneficiaries’ zip code of residence.^24^ We further categorized beneficiaries’ locality of residence based on metropolitan and nonmetropolitan area (including micropolitan, small town, and rural areas) using rural-urban commuting area codes that classify US census zip codes using measures of population density, urbanization, and daily commuting.^25^

### Statistical Analysis

**Figure 1.**
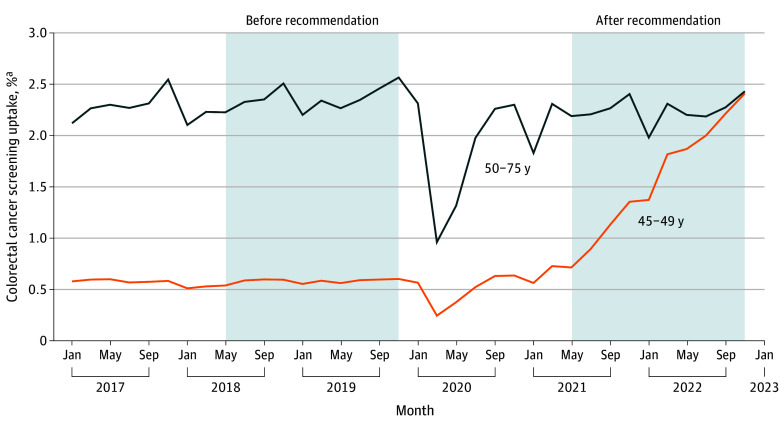
Bimonthly Colorectal Cancer (CRC) Screening Uptake Among Blue Cross Blue Shield (BCBS) Beneficiaries Aged 45 to 75 Years ^a^Screening uptake was calculated by dividing the number of BCBS beneficiaries in the age group who received CRC screening by the number who (1) had BCBS as the primary insurance for at least 12 months prior to the start of the period and remained enrolled through the end of the 2-month period and (2) did not receive CRC screening or related procedures in the 12 months preceding the 2-month period. Individuals screened multiple times were included to assess the overall utilization of each screening modality.

We categorized May 1, 2018, to December 31, 2019, the 20-month period preceding the USPSTF 2021 recommendation and the COVID-19 pandemic, as the prerecommendation period and the 20-month period from May 1, 2021, to December 31, 2022, following the USPSTF 2021 recommendation and the first 2 waves of the COVID-19 pandemic, as the postrecommendation period (Figure 1).^26,27^ We calculated the absolute and relative changes in screening uptake comparing the prerecommendation and postrecommendation periods.^28^ In addition, we used interrupted time-series analysis to assess the association between the USPSTF 2021 recommendation and CRC screening uptake.^29^ This method has been used to study the outcomes of large-scale policy interventions, including changes in USPSTF screening guidelines.^30,31,32^ Based on prior observation that changes in CRC screening are rapidly implemented following changes in guidelines,^7^ we assumed an immediate effect in our analysis.

To calculate rate of uptake since the recommendation was issued, we fit segmented regression to the series of bimonthly screening uptake, with parameters for intercept, baseline trend, and changes in level and trend comparing the prerecommendation and postrecommendation periods. The Durbin-Watson statistic was used to check for autocorrelation of residuals.^29^ If autocorrelation was observed, we used autoregressive integrated moving average (ARIMA) models that adjusted for autocorrelation and seasonality of the data.^29,33,34^ All analyses were performed using R, version 4.2.4 (R Project for Statistical Computing). All *P* values were from 2-sided tests, and results were deemed statistically significant at *P* < .05.

## Results

A total of 10 221 114 distinct beneficiaries aged 45 to 49 years were included in the study sample (mean [SD] age, 47.04 [1.41] years; 51.04% female; 48.96% male), with a mean (SD) of 3 304 879 (108 378) individuals eligible for CRC screening in every 2-month period. Of this cohort of beneficiaries, 1.15% were Asian, 2.65% were Black, 3.86% were Hispanic, 0.15% were Native American or Pacific Islander, 25.43% were White, and 66.76% had unknown race and ethnicity. As shown in Figure 1, mean (SD) CRC screening uptake among individuals aged 45 to 49 years was comparable before (January 1, 2017, to April 30, 2018; 0.57% [0.03%]) and after (May 1, 2018, to December 31, 2019; 0.58% [0.02%]) the ACS recommendation (*P* = .30). Screening uptake was only 0.24% in March and April 2020 (ie, onset of COVID-19) but increased steadily after May 2021 (ie, when the USPSTF recommendation was issued) (range, 0.71%-2.41%). Between November 1, 2021, and February 8, 2022, uptake increased slowly, possibly coinciding with the onset of the Omicron COVID-19 variant. In comparison, screening uptake among individuals aged 50 to 75 years remained relatively stable except during the period from March 1 to July 31, 2020. Furthermore, by December 31, 2022, screening uptake among individuals aged 45 to 49 years reached a level similar to that among individuals aged 50 to 75 years (2.37% and 2.40%, respectively).

**Figure 2.**
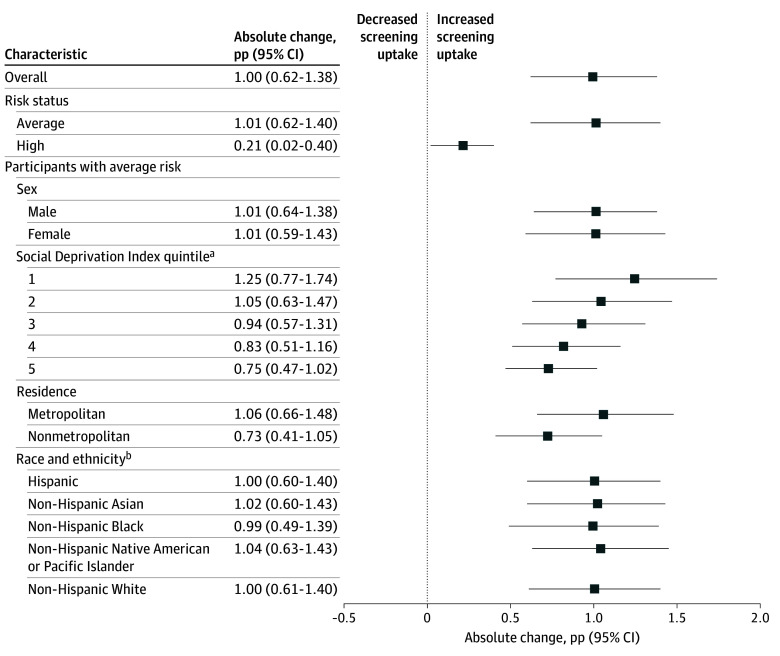
Absolute Change in Uptake of Screening for Colorectal Cancer Among Blue Cross Blue Shield Beneficiaries Aged 45 to 49 Years Comparing Prerecommendation and Postrecommendation Periods The prerecommendation period was from May 1, 2018, to December 31, 2019, and the postrecommendation period was from May 1, 2021, to December 31, 2022. pp Indicates percentage points. ^a^Quintile 1 represents the highest socioeconomic status. ^b^Race and ethnicity information was available for 35.2% and 32.1% of beneficiaries during the prerecommendation and postrecommendation periods, respectively.

**Figure 3.**
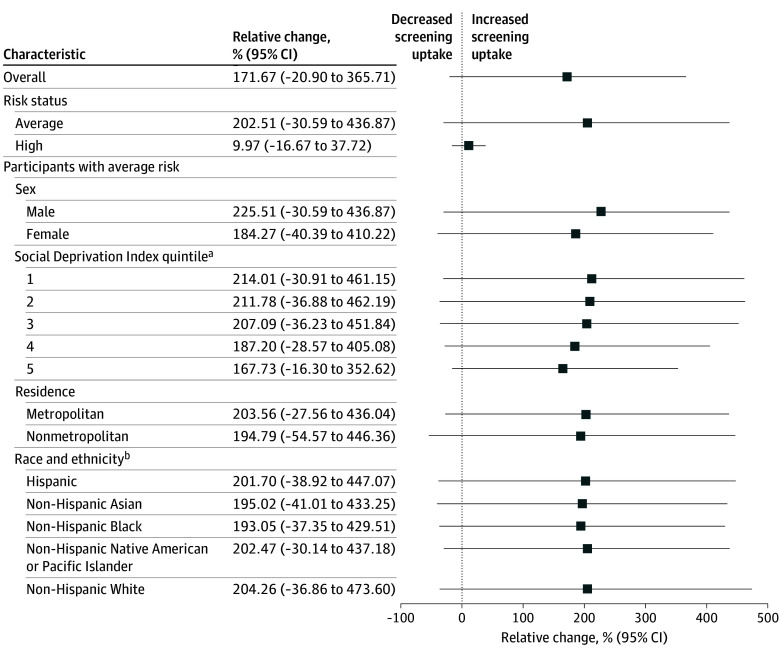
Relative Change in Uptake of Screening for Colorectal Cancer Among Blue Cross Blue Shield Beneficiaries Aged 45 to 49 Years Comparing Prerecommendation and Postrecommendation Periods The prerecommendation period was from May 1, 2018, to December 31, 2019, and the postrecommendation period was from May 1, 2021, to December 31, 2022. ^a^Quintile 1 represents the highest socioeconomic status. ^b^Race and ethnicity information was available for 35.2% and 32.1% of beneficiaries during the prerecommendation and postrecommendation periods, respectively.

The bimonthly mean (SD) number of beneficiaries aged 45 to 49 years who were eligible for CRC screening in the prerecommendation period was 3 385 126 (32 878) (Table). This number decreased to a mean (SD) of 3 194 246 (91 357) beneficiaries during the postrecommendation period. The bimonthly mean (SD) uptake of CRC screening increased significantly from 0.58% (0.02%) in the prerecommendation period to 1.58% (0.57%) in the postrecommendation period (*P* < .001). Overall, this represented an absolute change of 1.00 percentage points (95% CI, 0.62-1.38 percentage points) (Figure 2) but no significant relative change (171.67%; 95% CI, −20.90% to 365.71%) (Figure 3).

**Table.** Changes in CRC Screening Uptake Among Beneficiaries Aged 45 to 49 Years Comparing Prerecommendation and Postrecommendation Periods^a^

Characteristic	Bimonthly frequency of beneficiaries enrolled, mean (SD), No.		Bimonthly uptake of CRC screening, mean (SD), %		*P* value		
	Preperiod	Postperiod	Preperiod	Postperiod	Preperiod vs postperiod	Subgroups in preperiod	Subgroups in postperiod
Overall	3 385 126 (32 878)	3 194 246 (91 357)	0.58 (0.02)	1.58 (0.57)	<.001	NA	NA
Risk status							
Average	3 213 935 (31 508)	2 923 327 (105 716)	0.50 (0.02)	1.51 (0.59)	<.001	<.001	<.001
High	171 191 (25 201)	270 919 (14 948)	2.12 (0.11)	2.33 (0.27)	<.001	Reference	Reference
**Average-risk participants**							
Sex							
Female	1 613 628 (17 128)	1 463 131 (56 450)	0.55 (0.02)	1.56 (0.63)	<.001	Reference	Reference
Male	1 600 308 (15 252)	1 460 196 (49 300)	0.45 (0.02)	1.46 (0.56)	<.001	<.001	.69
Social Deprivation Index, quintile^b^							
1	888 305 (8248)	803 051 (34 482)	0.59 (0.02)	1.84 (0.73)	<.001	Reference	Reference
2	729 188 (7544)	660 800 (24 283)	0.50 (0.02)	1.55 (0.63)	<.001	<.001	.36
3	653 074 (6094)	591 746 (21 013)	0.45 (0.02)	1.39 (0.56)	<.001	<.001	.14
4	547 018 (5536)	498 909 (16 113)	0.45 (0.02)	1.28 (0.49)	<.001	<.001	.06
5	348 038 (5135)	327 849 (7991)	0.44 (0.01)	1.19 (0.42)	<.001	<.001	.02
Locality							
Metropolitan	2 633 104 (25 197)	2 405 378 (89 340)	0.53 (0.02)	1.59 (0.62)	<.001	Reference	Reference
Nonmetropolitan	564 877 (6214)	503 313 (15 929)	0.38 (0.02)	1.11 (0.48)	<.001	<.001	.06
Race and ethnicity^c^							
Hispanic	133 633 (1818)	106 438 (5060)	0.50 (0.03)	1.50 (0.61)	<.001	<.001	.99
Non-Hispanic Asian	40 173 (357)	32 375 (1474)	0.52 (0.02)	1.54 (0.63)	<.001	<.001	.90
Non-Hispanic Black	88 409 (834)	75 119 (1139)	0.51 (0.04)	1.50 (0.60)	<.001	<.001	.99
Non-Hispanic Native American or Pacific Islander	5388 (92)	4237 (137)	0.51 (0.08)	1.55 (0.62)	<.001	<.001	.86
Non-Hispanic White	864 698 (8827)	721 076 (20 700)	0.50 (0.02)	1.50 (0.59)	<.001	Reference	Reference
Unknown	2 191 724 (23 162)	2 163 379 (67 380)	0.58 (0.02)	1.58 (0.57)	<.001	<.001	.76

Abbreviations: CRC, colorectal cancer; NA, not applicable.

^a^ The prerecommendation period was from May 1, 2018, to December 31, 2019, and the postrecommendation period was from May 1, 2021, to December 31, 2022.

^b^ Area-level social deprivation index was calculated based on 7 demographic characteristics: the proportion living in poverty, with less than 12 years of education, of single-parent households, living in rented housing units, living in overcrowded housing units, of households without a car, and of unemployed adults younger than 65 years. Quintile 1 represents the highest socioeconomic status.

^c^ Race and ethnicity information was available for 35.2% and 32.1% of beneficiaries during the prerecommendation and postrecommendation periods, respectively.

Stratified analysis by risk status indicated that both average- and high-risk beneficiaries experienced significant increases in screening between the prerecommendation and postrecommendation periods. A bimonthly mean (SD) of 3 213 935 (31 508) and 2 923 327 (105 716) beneficiaries were classified as average risk in the prerecommendation and postrecommendation periods, respectively (Table). Among average-risk beneficiaries, mean (SD) screening uptake increased from 0.50% (0.02%) to 1.51% (0.59%) between the 2 periods (*P* < .001). There were a mean (SD) of 171 191 (25 201) and 270 919 (14 948) beneficiaries classified as having high risk for CRC during the prerecommendation and postrecommendation periods, respectively. In this high-risk group, mean (SD) screening uptake changed from 2.12% (0.11%) to 2.33% (0.27%) (*P* < .001). Although high-risk beneficiaries had significantly higher screening uptake than average-risk beneficiaries in both periods, average-risk beneficiaries experienced a higher absolute change in screening (1.01 percentage points [95% CI, 0.62-1.40 percentage points] vs 0.21 percentage points [95% CI, 0.02-0.40 percentage points]); however, average-risk beneficiaries had no significant relative change in screening (202.51%; 95% CI, −30.59% to 436.87%) (Figure 2 and Figure 3).

In the prerecommendation period, colonoscopy accounted for 41.3% of all screening tests followed by FIT (32.2%) and fecal occult blood test (24.6%) (eFigure and eTable 3 in Supplement 1). Stool DNA tests, flexible sigmoidoscopy, double-contrast barium enemas, and computed tomography colonography comprised the remaining modalities. In the postrecommendation period, colonoscopy remained the most common screening modality (52.7%) followed by stool DNA test (25.0%) and FIT (16.2%).

Females with average risk had significantly higher uptake of overall screening in the postrecommendation period compared with the prerecommendation period (1.56% vs 0.55%; *P* < .001). Similarly, males with average risk had significantly higher uptake of overall screening in the postrecommendation period compared with the prerecommendation period (1.46% vs 0.45%; *P* < .001). Postrecommendation screening uptake did not differ significantly between females and males (1.56% vs 0.45%; *P* = .69). In the postrecommendation period, use of colonoscopy was 0.65% among males compared with 0.60% among females (*P* = .74), and use of stool DNA test was 0.31% among females compared with 0.26% among males (*P* = .57) (Table and eTable 3 in Supplement 1).

After the recommendation was issued in May 2021, uptake of screening was significantly higher among average-risk beneficiaries residing in areas with SDI in the first quintile (ie, highest SES) compared with beneficiaries in the fifth quintile (ie, lowest SES) (1.84% vs 1.19%; *P* = .02) (Table). Beneficiaries residing in areas in the first SDI quintile also experienced the largest absolute change in screening between the prerecommendation and postrecommendation periods (1.25 percentage points; 95% CI, 0.77-1.74 percentage points), although there was no relative change in screening (214.01%; 95% CI, −30.91% to 461.15%) (Figure 2 and Figure 3). In comparison, beneficiaries residing in areas in the fifth quintile of SDI experienced an absolute change in screening uptake of 0.75 percentage points (95% CI, 0.47-1.02 percentage points) but no relative change (167.73%; 95% CI, −16.30% to 352.62%) between the 2 periods. Stratified analysis by screening modality similarly showed highest and lowest screening in individuals residing in areas in the first and fifth SDI quintiles, respectively (eTable 3 in Supplement 1).

Overall, average-risk beneficiaries residing in metropolitan areas had significantly higher uptake of screening during both periods compared with beneficiaries residing in nonmetropolitan areas (0.53% vs 0.38% prerecommendation [*P* < .001] and 1.59% vs 1.11% postrecommendation [*P* = .06]) (Table). However, beneficiaries in both metropolitan and nonmetropolitan areas experienced a significant increase in screening between the 2 periods. Among average-risk beneficiaries residing in metropolitan areas, mean (SD) uptake of screening increased from 0.53% (0.02%) to 1.59% (0.62%), but there was no significant relative change (203.56%; 95% CI, −27.56% to 436.04%) (Figure 2 and Figure 3). Among beneficiaries residing in nonmetropolitan areas, mean (SD) uptake of screening increased from 0.38% (0.02%) to 1.11% (0.48%) but with no significant relative change (194.79%; 95% CI, −54.57% to 446.36%). Stratified analysis by screening modality showed higher, though not significantly different, use of colonoscopy between metropolitan and nonmetropolitan areas in the postrecommendation period (0.66% vs 0.45%; *P* = .10). Similarly, use of stool DNA tests did not vary significantly between metropolitan and nonmetropolitan areas in the postrecommendation period (0.29% vs 0.25%; *P* = .59) (eTable 3 in Supplement 1).

**Figure 4.**
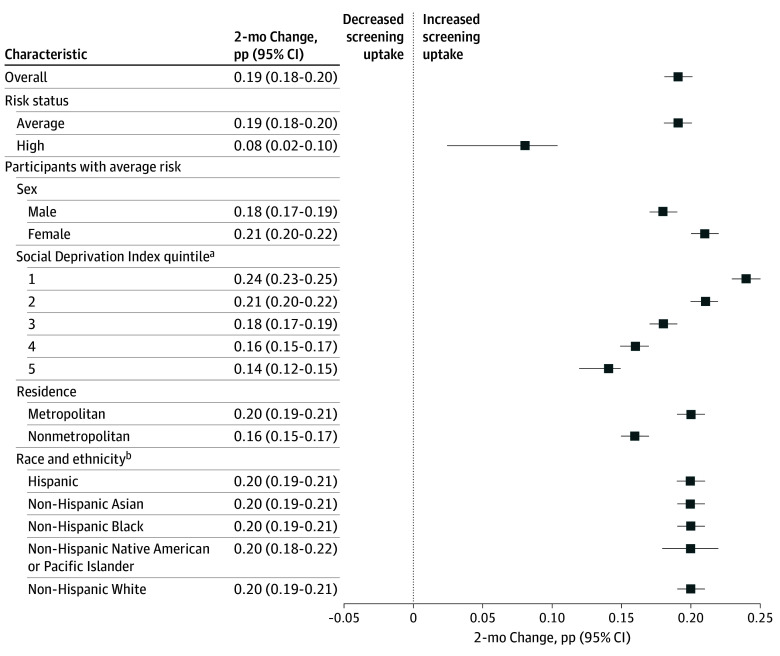
Results From Stratified Autoregressive Integrated Moving Average Models Assessing 2-Month Change in Rate of Postrecommendation Colorectal Cancer Screening Uptake Data are from May 1, 2021, to December 31, 2022, for Blue Cross Blue Shield beneficiaries aged 45 to 49 years. pp Indicates percentage points. ^a^Quintile 1 represents the highest socioeconomic status. ^b^Race and ethnicity information was available for 35.2% and 32.1% of beneficiaries during the prerecommendation and postrecommendation periods, respectively.

Results from stratified ARIMA models assessing the rate of screening uptake since the recommendation was issued indicated an increasing rate of screening uptake among beneficiaries aged 45 to 49 years overall and across subgroups. After the recommendation was issued in May 2021, uptake of CRC screening in the overall cohort increased 0.19 percentage points (95% CI, 0.18-0.20 percentage points) every 2 months (Figure 4). Among average-risk beneficiaries, screening uptake overall increased 0.19 percentage points (95% CI, 0.18-0.20 percentage points) every 2 months. Beneficiaries residing in the areas in the first SDI quintile experienced the highest increase in trend followed by beneficiaries in the second to fifth quintiles. Screening uptake increased 0.24 percentage points (95% CI, 0.23-0.25 percentage points) every 2 months among those residing in the first SDI quintile areas and 0.14 percentage points (95% CI, 0.12-0.15 percentage points) every 2 months among those residing in the fifth SDI quintile areas. Screening uptake among metropolitan area residents increased 0.20 percentage points (95% CI, 0.19-0.21 percentage points) every 2 months, whereas nonmetropolitan area residents experienced an increase of 0.16 percentage points (95% CI, 0.15-0.17 percentage points) in screening uptake every 2 months.

Overall, 13.9% of beneficiaries aged 45 to 49 years during the postrecommendation period received CRC screening before reaching the age of 50 years (eTable 4 in Supplement 1). A total of 11.5% and 37.1% of average- and high-risk beneficiaries aged 45 to 49 years during the postrecommendation period, respectively, received CRC screening.

### Exploratory Analysis

In the subgroup of average-risk beneficiaries with imputed data on race and ethnicity (35.2% and 32.1% of beneficiaries during the prerecommendation and postrecommendation periods, respectively), each racial and ethnic group experienced a significant increase in screening between the 2 periods. Among White beneficiaries, mean (SD) uptake of screening increased from 0.50% (0.02%) to 1.50% (0.59%) (*P* < .001) (Figure 2). Among Black beneficiaries, mean (SD) uptake increased similarly from 0.51% (0.04%) to 1.50% (0.60%) (*P* < .001). Mean (SD) screening uptake among Asian, Native American or Pacific Islander, and Hispanic beneficiaries ranged from 0.50% (0.03%) to 0.52% (0.02%) in the prerecommendation period and from 1.50% (0.61%) to 1.55% (0.62%) in the postrecommendation period (*P* < .001 for both). No significant difference in screening uptake was observed between White beneficiaries and other beneficiaries, including those with unknown race and ethnicity, during the postrecommendation period (Table). Stratified analysis by screening modality showed no significant difference in colonoscopy and stool DNA test use based on race and ethnicity (eTable 3 in Supplement 1). Furthermore, stratified analysis evaluating differences in screening uptake by SDI within each racial and ethnic group showed that individuals residing in areas in the first and fifth SDI quintile had the highest and lowest uptake of CRC screening, respectively (eTable 5 in Supplement 1).

## Discussion

Our results indicated that CRC screening uptake among individuals aged 45 to 49 years significantly increased following the May 2021 USPSTF recommendation. Although CRC screening was expectedly higher among high-risk individuals, the 2-fold increase in screening among average-risk beneficiaries suggests a shift in the screening paradigm of this newly included age group. Although statistically significantly different, screening uptake was relatively low in all subgroups in the prerecommendation period. However, in the postrecommendation period, widening disparities based on SDI and locality were observed. These disparities persisted among individuals with known or unknown race and ethnicity information.

We observed a similar uptake of CRC screening in beneficiaries 45 to 49 years of age preceding and following the ACS recommendation in 2018, which may be attributable to the ACS recommendation not stipulating any insurance mandate. Although a prior study identified significant increases in screening following the 2018 ACS recommendation,^7^ our study in a large cohort of privately insured individuals did not find evidence of increased screening following this recommendation. However, the shift in insurance coverage due to the USPSTF 2021 recommendation was significantly associated with changes in screening practices among beneficiaries aged 45 to 49 years. Additionally, during the first wave of the COVID-19 pandemic (March and April 2020), almost all screening was eliminated in this age group; however, screening recovered to prepandemic levels in September 2020, and screening uptake remained relatively unchanged during the second and third waves of the pandemic. Overall, mean frequency of eligible beneficiaries was lower in the postrecommendation period compared with the prerecommendation period (3.2 million vs 3.4 million). This may be due to a decrease in job-based insurance coverage following the COVID-19 pandemic.^35^

Insurance status is a key factor contributing to disparities in screening.^4^ All individuals in the study population had private insurance through BCBS, but the observed disparity in CRC screening based on neighborhood SDI and locality highlight the persistent role of socioeconomic and community-level factors that contribute to gaps in screening among subgroups of beneficiaries. The mechanisms behind these persistent disparities have not been fully elucidated, although factors such as perception of low health care quality, no routine checkup within 2 years, and perceived discrimination may have exacerbated these disparities.^11,36^

The 3-fold increase in screening uptake among average-risk individuals aged 45 to 49 years reflects an accomplishment, yet evidence of widening disparities based on SDI and locality indicate that population subgroups may not be benefiting equally from this change in CRC screening recommendation. Furthermore, given that only 11.5% of average-risk individuals aged 45 to 49 years during the postrecommendation period received CRC screening before the age of 50 years, targeted initiatives to improve screening in this age group are warranted to reach the national goal of screening 80% of the population in every community.

### Limitations

There are limitations to our study. Data on race and ethnicity were only available for a subset of the population, undermining our ability to thoroughly evaluate the potential influence of these demographic factors. Although we analyzed SDI and locality as factors that represent multiple domains of the social and physical environment, prior studies showed that SES alone does not capture the pervasive association of race with health outcomes.^37,38^ As BCBS is currently updating race and ethnicity information for a larger proportion of the study cohort, future studies may continue to evaluate changes in screening patterns among diverse population subgroups. We did not have specific information on the type of coverage provided by each beneficiary’s insurance plan, which may have impacted their screening uptake in the prerecommendation period. Since the USPSTF recommendation mandates all insurance programs to cover the cost of CRC screening among individuals aged 45 to 49 years, potential differences in uptake based on type of insurance may have been alleviated in the postrecommendation period. Although BCBS is the largest provider of commercial health insurance, the study cohort may not be fully representative of the general US population because BCBS beneficiaries tend to be younger and more socioeconomically advantaged with employer-based insurance. Nevertheless, it has been demonstrated that patterns of cancer care in the BCBS population are generalizable to the Medicare population.^39^ As the focus of our analysis was CRC screening among individuals 45 to 49 years of age, BCBS is a great resource. Additionally, there are substantial SES variations among BCBS beneficiaries, which allowed for stratified analysis to assess disparity in CRC screening.

## Conclusions

This cohort study of 10 221 114 individuals found that CRC screening uptake increased significantly among those aged 45 to 49 years after the USPSTF issued its qualified recommendation in May 2021 that encouraged screening in this age group. A significant increasing rate was observed overall and in racial and ethnic, SES, and metropolitan and nonmetropolitan locality subgroups, but disparities in screening were observed based on socioeconomic status and locality, underlining an urgent need to improve CRC screening uptake for all.

## Notes

Supplement 1. **eMethods**. Events of Interest and Determination of High and Average Risk Status **eTable 1.** Numerators and Denominators Used to Calculate Colorectal Cancer Screening Uptake Among BCBS Beneficiaries Aged 45 to 49 Years **eTable 2.** Codes Used to Identify Colorectal Cancer Screening–Related Procedures, High-Risk Individuals, and Diagnostic Procedures **eTable 3.** Changes in Bimonthly Colonoscopy and Stool DNA Tests Before and After the USPSTF Recommendation Among Individuals at Average Risk Aged 45 to 49 Years and Insured by BCBS **eTable 4.** Percentage of BCBS Beneficiaries Aged 45 to 49 Years Between May 2021 and December 2022 Who Received Any Screening for Colorectal Cancer Before Age 50 Years **eTable 5.** CRC Screening Uptake by Social Deprivation Index Within Each Race and Ethnicity Group Among BCBS Beneficiaries Aged 45 to 49 Years During the Postrecommendation Period **eFigure.** Changes in Colorectal Cancer Screening Uptake Among BCBS Axis Beneficiaries Aged 45 to 49 Years With Average Risk of Colorectal Cancer, Stratified by Screening Modality
eReferences

Supplement 2.
Data Sharing Statement
