# Supplementary material for: USPSTF Colorectal Cancer Screening Recommendation and Uptake for Individuals Aged 45 to 49 Years
Source: JAMA Netw Open. 2024 Oct 3;7(10):e2436358. doi: 10.1001/jamanetworkopen.2024.36358 (PMC11450516; doi:10.1001/jamanetworkopen.2024.36358)
Supplement: Supplement 1. — eMethods. Events of Interest and Determination of High and Average Risk Status eTable 1. Numerators and Denominators Used to Calculate Colorectal Cancer Screening Uptake Among BCBS Beneficiaries Aged 45 to 49 Years eTable 2. Codes Used to Identify Colorectal Cancer Screening–Related Procedures, High-Risk Individuals, and Diagnostic Procedures eTable 3. Changes in Bimonthly Colonoscopy and Stool DNA Tests Before and After the USPSTF Recommendation Among Individuals at Average Risk Aged 45 to 49 Years and Insured by BCBS eTable 4. Percentage of BCBS Beneficiaries Aged 45 to 49 Years Between May 2021 and December 2022 Who Received Any Screening for Colorectal Cancer Before Age 50 Years eTable 5. CRC Screening Uptake by Social Deprivation Index Within Each Race and Ethnicity Group Among BCBS Beneficiaries Aged 45 to 49 Years During the Postrecommendation Period eFigure. Changes in Colorectal Cancer Screening Uptake Among BCBS Axis Beneficiaries Aged 45 to 49 Years With Average Risk of Colorectal Cancer, Stratified by Screening Modality eReferences [file jamanetwopen-e2436358-s001.pdf]

Supplementary Online Content

Siddique S, Wang R, Yasin F, et al. USPSTF colorectal cancer screening recommendation and uptake for individuals aged 45 to 49 years. *JAMA Netw Open*. 2024;7(10):e2436358. doi:10.1001/jamanetworkopen.2024.36358

eMethods. Events of Interest and Determination of High and Average Risk Status

eTable 1. Numerators and Denominators Used to Calculate Colorectal Cancer Screening Uptake Among BCBS Beneficiaries Aged 45 to 49 Years

eTable 2. Codes Used to Identify Colorectal Cancer Screening–Related Procedures, High-Risk Individuals, and Diagnostic Procedures

eTable 3. Changes in Bimonthly Colonoscopy and Stool DNA Tests Before and After the USPSTF Recommendation Among Individuals at Average Risk Aged 45 to 49 Years and Insured by BCBS

eTable 4. Percentage of BCBS Beneficiaries Aged 45 to 49 Years Between May 2021 and December 2022 Who Received Any Screening for Colorectal Cancer Before Age 50 Years

eTable 5. CRC Screening Uptake by Social Deprivation Index Within Each Race and Ethnicity Group Among BCBS Beneficiaries Aged 45 to 49 Years During the Postrecommendation Period

eFigure. Changes in Colorectal Cancer Screening Uptake Among BCBS Axis Beneficiaries Aged 45 to 49 Years With Average Risk of Colorectal Cancer, Stratified by Screening Modality

eReferences

This supplementary material has been provided by the authors to give readers additional information about their work.

© 2024 Siddique S et al. *JAMA Network Open*.

eMethods: Events of interest and determination of high and average risk status

Events of Interest

Several events which might impact CRC screening, including the COVID-19 pandemic, occurred in our study period: 1) ACS recommendation encouraging CRC screening among individuals aged ≥ 45 years (May 2018); 2) first wave of COVID-19 outbreak (March – April 2020); 3) second wave of COVID-19 outbreak (January – February 2021); 4) USPSTF recommendation for screening among individuals aged ≥ 45 years (May 2021); and 5) third wave of COVID-19 outbreak (January – March 2022).[1-3] Upon consideration of these critical events, the USPSTF 2021 recommendation was chosen as the primary event of interest due to its implications on insurance coverage for CRC screening. Under section 2713 of the Public Health Service Act, non-grandfathered group health plans and health insurance coverage offered in the individual and group market are mandated to cover the cost of preventive services that have a rating of "A" or "B" in the current recommendations of the USPSTF.[4]

Risk Status

As the USPSTF 2021 recommendation for starting CRC screening at the age of 45 years applied to individuals who are at average risk for CRC, we assessed an individual's CRC risk using claims data. High-risk individuals were identified based on the following criteria: history of CRC, colorectal polyp, irritable bowel disease, family history of gastrointestinal cancer, and genetic susceptibility to other malignant neoplasms.[5] (eTable 2) Any individual who was not identified as high-risk was considered to be average-risk. Screening recommendations for high-risk individuals vary based on which condition qualifies them as "high risk" and the age at diagnosis of the relative who had the related condition. For example, in 2017 the US Multi-Society Task Force on Colorectal Cancer recommended that high risk individuals, such as persons with a family history of CRC are recommended to undergo screening by colonoscopy

© 2024 Siddique S et al. *JAMA Network Open*.

every 5 years, beginning 10 years before the age at diagnosis of the youngest affected relative or age 40, whichever is earlier.[6,7] Due to differences in screening recommendations for high-risk individuals, our analysis primarily focused on individuals with average-risk of CRC.

© 2024 Siddique S et al. *JAMA Network Open*.

eTable 1. Numerators and denominators used to calculate colorectal cancer screening uptake among BCBS beneficiaries aged 45-49 years

Month	Total Number of Eligible Individuals	Total Number of Individuals Receiving Screening	Uptake (% Screened)
2017/01	3,180,244	18,389	0.58%
2017/03	3,206,171	19,103	0.60%
2017/05	3,213,109	19,259	0.60%
2017/07	3,217,440	18,268	0.57%
2017/09	3,220,400	18,485	0.57%
2017/11	3,218,827	18,762	0.58%
2018/01	3,339,576	17,054	0.51%
2018/03	3,358,315	17,782	0.53%
2018/05	3,353,559	18,046	0.54%
2018/07	3,351,205	19,693	0.59%
2018/09	3,357,619	20,058	0.60%
2018/11	3,353,720	19,944	0.59%
2019/01	3,439,980	19,036	0.55%
2019/03	3,430,020	20,058	0.58%
2019/05	3,410,251	19,151	0.56%
2019/07	3,395,991	20,050	0.59%
2019/09	3,379,954	20,154	0.60%
2019/11	3,378,961	20,335	0.60%
2020/01	3,408,317	19,274	0.57%
2020/03	3,411,027	8,340	0.24%
2020/05	3,416,958	12,826	0.38%
2020/07	3,411,144	17,822	0.52%
2020/09	3,389,059	21,379	0.63%
2020/11	3,371,472	21,421	0.64%
2021/01	3,417,448	19,239	0.56%
2021/03	3,402,424	24,743	0.73%
2021/05	3,349,652	23,920	0.71%
2021/07	3,301,388	29,517	0.89%
2021/09	3,253,950	36,812	1.13%
2021/11	3,220,288	43,607	1.35%
2022/01	3,211,754	44,069	1.37%
2022/03	3,185,021	57,876	1.82%
2022/05	3,144,605	58,761	1.87%
2022/07	3,115,020	62,280	2.00%
2022/09	3,089,911	68,368	2.21%
2022/11	3,070,866	73,996	2.41%

© 2024 Siddique S et al. *JAMA Network Open*.

eTable 2. Codes used to identify colorectal cancer screening related procedures, high-risk individuals, and diagnostic procedures

Codes used to identify colorectal cancer screening		
Procedure Name	Code Type	Code
Fecal occult blood test	CPT/HCPCS	G0327, G0107, 82270, 82271, 82272, 82273
Fecal immunochemical test	CPT/HCPCS	G0328, 82274
Stool DNA test (Cologuard®)	CPT/HCPCS	81528, G0464 , S3890 ALSO BY NPI
Flexible sigmoidoscopy	CPT/HCPCS	G0104, G6022, 45300, 45303, 45305, 45307, 45308, 45309, 45315, 45317, 45320, 45321, 45327, 45330, 45331, 45332, 45333, 45334, 45335, 45337,45338, 45339, 45340, 45341, 45342, 45345, 45346, 45349
Double-contrast barium enema	CPT/HCPCS	G0106, G0120, G0122, 74270, 74280
Colonoscopy	CPT/HCPCS	G0105, G0121, G6024, 44388-44394, 44397, 45355, 45378, 45379, 45380, 45381, 45382, 45383, 45384, 45385, 45386, 45387, 45388, 45390, 45391, 45392
CT colonography	CPT/HCPCS	74263, 0066T
**Codes used to identify high risk individuals**		
High-risk Condition	Code Type	Codes
Colorectal polyp	ICD-9-CM	2113, 2114, 5690, 5564, 56949, 20950–20957
	ICD-10-CM	D120–D128, K5140, K51411–K51414, K51418, K51419, K620, K621, K635, D3A020–D3A026, D3A029
History of colorectal polyp	ICD-9-CM	V1272
	ICD-10-CM	Z86010
Colorectal cancer	ICD-9-CM	1530–1541, 1548, 1975, 2303, 2304, 20910–20917
	ICD-10-CM	C180–C189, C19, C20, C212, C218, C785, C7A020–C7A026, C7A029, D0140, D0149, D010–D012
History of colorectal cancer	ICD-9-CM	V1005, V1006
	ICD-10-CM	Z85030, Z85038, Z85040, Z85048, Z8509
Family history of gastrointestinal cancer	ICD-9-CM	V160
	ICD-10-CM	Z800
Irritable bowel disease	ICD-9-CM	5550–5552, 5559–5563, 5565, 5566, 5568, 5569

© 2024 Siddique S et al. *JAMA Network Open*.

	ICD-10-CM	K5000, K50011–K50014, K50018, K50019, K5010, K5011, K50111–K50114, K50118, K50119, K508, K5080, K50811–K50814, K50818, K50819, K5090, K50911–K50914, K50918, K50919, K5100, K51011–K51014, K51018, K51019, K5120, K51211–K51214, K51218, K51219, K513, K5130, K51311–K51314, K51318, K51319, K5150, K51511–K51514, K51518, K51519, K5180, K51811–K51814, K51818, K51819, K5190, K51911–K51914, K51918, K51919, K5289
Genetic susceptibility to other malignant neoplasm	ICD-9-CM	V8409
	ICD-10-CM	Z1509

Codes used to distinguish diagnostic procedures from screening related procedures

Condition	Code Type	Code
Abdominal Pain	ICD-9-CM	787.3, 789.6, 789
	ICD-10-CM	R14, R10.81, R10.82
Altered bowel habits	ICD-9-CM	564,787
	ICD-10-CM	K52.29, K52.89, K58, K59 EXCEPT K59.31, R11.0, R11.10, R11.11-2, R11.14-5, R12-5, R19.11-12, R19.15, R19.4-5, R19.7, R19.8
Gastrointestinal bleeding	ICD-9-CM	578
	ICD-10-CM	K92.0-2
Positive fecal occult blood test	ICD-9-CM	792.1
	ICD-10-CM	R19.5
Weight loss	ICD-9-CM	783.2
	ICD-10-CM	R63.4, R63.6
Iron deficiency anemia	ICD-9-CM	280
	ICD-10-CM	D50
Anemia unspecified	ICD-9-CM	285.9
	ICD-10-CM	D64.9
Sigmoidoscopy	ICD-9-CM	45.24, 48.21-4
	ICD-10-CM	0DJD8ZZ, 0D9P3ZX

© 2024 Siddique S et al. *JAMA Network Open*.

eTable 3. Changes in Bimonthly Colonoscopy and Stool DNA Tests Before and After the USPSTF Recommendation (May 2018 - December 2019 vs. May 2021 - December 2022) Among Average-Risk Individuals Aged 45-49 years and Insured by Blue Cross Blue Shield

	**Colonoscopy**							
	Pre-Recommendation (41.3% of all screening)			Post- Recommendation (52.7% of all screening)			P-value Comparing Pre- vs Post-Onset	Bimonthly Percentage Change (%, 95% CI) [b]
	Mean Frequency of Beneficiaries Screened (SD)	Mean CRC Screening (%, SD)	P-value Comparing Subgroups	Mean Frequency of Beneficiaries Screened (SD)	Mean CRC Screening (%, SD)	P-value Comparing Subgroups		
**Overall**	6,149 (723)	0.19 (0.02)		18,166 (11,455)	0.62 (0.42)		<0.001	0.124 (0.118, 0.130)
**Sex**								
Females	2,828 (368)	0.18 (0.02)	Ref	8,701 (5,731)	0.60 (0.42)	Ref	<0.001	0.127 (0.120, 0.133)
Males	3,321 (368)	0.21 (0.02)	<0.001	9,465 (5,734)	0.65 (0.42)	0.74	<0.001	0.123 (0.115, 0.128)
**Social Deprivation Index**								
1st (highest SES)	2,081 (283)	0.24 (0.03)	Ref	6,406 (4,083)	0.80 (0.51)	Ref	<0.001	0.162 (0.154, 0.170)
2nd	1,420 (153)	0.20 (0.02)	<0.001	4,191 (2,683)	0.64 (0.43)	0.37	<0.001	0.130 (0.124, 0.136)
3rd	1,143 (119)	0.18 (0.02)	<0.001	3,326 (2,127)	0.56 (0.38)	0.17	<0.001	0.114 (0.109, 0.120)
4th	884 (106)	0.16 (0.02)	<0.001	2,475 (1,524)	0.50 (0.33)	0.07	<0.001	0.097 (0.094, 0.100)
5th (lowest SES)	522 (75)	0.15 (0.02)	<0.001	1,527 (906)	0.47 (0.29)	0.04	<0.001	0.083 (0.078, 0.088)
**Locality**								
Metropolitan	5,187 (635)	0.20 (0.02)	Ref	15,913 (10,015)	0.66 (0.45)	Ref	<0.001	0.132 (0.125, 0.138)
Non-Metropolitan	926 (102)	0.17 (0.02)	<0.001	2,156 (1,392)	0.43 (0.29)	0.1	<0.001	0.090 (0.085, 0.095)
**Race and Ethnicity [a]**								
Hispanic	253 (29)	0.19 (0.02)	0.75	652 (412)	0.62 (0.42)	0.98	<0.001	0.128 (0.123, 0.133)
NH Asian	80 (11)	0.20 (0.03)	0.31	208 (130)	0.64 (0.44)	0.87	<0.001	0.134 (0.131, 0.138)
NH Black	170 (29)	0.19 (0.03)	0.88	472 (312)	0.63 (0.43)	0.96	<0.001	0.123 (0.120, 0.135)
NH Native American	10 (2)	0.19 (0.04)	0.52	26 (18)	0.62 (0.44)	0.99	<0.001	0.122 (0.100, 0.143)
NH White	1,657 (183)	0.19 (0.02)	Ref	4,464 (2,829)	0.62 (0.42)	Ref	<0.001	0.123 (0.118, 0.129)
Unknown	3,977 (491)	0.19 (0.02)	0.99	12,344 (7,759)	0.63 (0.42)	0.63	<0.001	0.124 (0.120, 0.128)
	**Stool DNA Test**							

© 2024 Siddique S et al. *JAMA Network Open*.

	Pre- Recommendation (1.8% of all tests)			Post- Recommendation (26.0% of all tests)			P-value Comparing Pre- vs Post-Onset	Bimonthly Percentage Change (%, 95% CI) [b]
	Mean Frequency of Beneficiaries Screened (SD)	Mean CRC Screening (%, SD)	P-value Comparing Subgroups	Mean Frequency of Beneficiaries Screened (SD)	Mean CRC Screening (%, SD)	P-value Comparing Subgroups		
**Overall**	217 (286)	0.01 (0.01)		8,154 (6,061)	0.28 (0.22)		<0.001	0.061 (0.057, 0.063)
**Sex**								
Females	111 (146)	0.01 (0.01)	Ref	4,410 (3,393)	0.31 (0.26)	Ref	<0.001	0.069 (0.067, 0.071)
Males	105 (140)	0.01 (0.01)	0.91	3,744 (2,675)	0.26 (0.19)	0.57	<0.001	0.052 (0.048, 0.055)
**Social Deprivation Index**								
1st (highest SES)	67 (88)	0.01 (0.01)	Ref	2,650 (1,920)	0.33 (0.26)	Ref	<0.001	0.070 (0.067, 0.074)
2nd	53 (68)	0.01 (0.01)	<0.001	1,955 (1,477)	0.30 (0.24)	0.7	<0.001	0.066 (0.062, 0.070)
3rd	44 (57)	0.01 (0.01)	0.78	1,577 (1,210)	0.27 (0.22)	0.46	<0.001	0.059 (0.057, 0.061)
4th	34 (46)	0.01 (0.01)	0.67	1,235 (921)	0.25 (0.19)	0.31	<0.001	0.053 (0.051, 0.056)
5th (lowest SES)	17 (25)	0.01 (0.01)	0.32	638 (468)	0.20 (0.15)	0.08	<0.001	0.041 (0.038, 0.043)
**Locality**								
Metropolitan	178 (238)	0.01 (0.01)	Ref	6,885 (5,045)	0.29 (0.22)	Ref	<0.001	0.061 (0.058, 0.064)
Non-Metropolitan	38 (47)	0.01 (0.01)	0.98	1,228 (993)	0.25 (0.21)	0.59	<0.001	0.060 (0.056, 0.064)
**Race and Ethnicity [a]**								
Hispanic	9 (12)	0.01 (0.01)	0.85	304 (235)	0.29 (0.24)	0.92	<0.001	0.067 (0.063, 0.070)
NH Asian	4 (5)	0.01 (0.01)	0.45	95 (72)	0.30 (0.24)	0.85	<0.001	0.064 (0.056, 0.072)
NH Black	6 (8)	0.01 (0.01)	0.9	205 (157)	0.27 (0.21)	0.92	<0.001	0.060 (0.058, 0.061)
NH Native American	1 (1)	0.01 (0.02)	0.92	14 (11)	0.33 (0.26)	0.57	<0.01	0.066 (0.058, 0.074)
NH White	56 (74)	0.01 (0.01)	Ref	2,023 (1,504)	0.28 (0.22)	Ref	<0.001	0.061 (0.058, 0.063)
Unknown	141 (187)	0.01 (0.01)	0.94	5,512 (4,087)	0.28 (0.22)	0.28	<0.001	0.060 (0.057, 0.063)

Abbreviations: CRC = Colorectal Cancer, NH = non-Hispanic, SD = Standard Deviation, SES = Socioeconomic Status

[a] Race and ethnicity information was available for 35.2% and 32.1% of beneficiaries during the pre-recommendation and post-recommendation periods, respectively.

[b] Results from autoregressive integrated moving average models estimating percentage change in screening uptake every 2 months during the post-recommendation period.

© 2024 Siddique S et al. *JAMA Network Open*.

eTable 4. Percent of BCBS beneficiaries who were aged 45-49 years between 5/2021 to 12/2022 and received any screening for colorectal cancer before age 50

	Total	Ever Screened	Screening Uptake (%)
Any Risk [a]	5,213,471	722,823	13.90%
Average Risk [b]	4,766,127	546,957	11.50%
High Risk [c]	510,623	189,374	37.10%

[a] Includes individuals aged 45-49 years who ever had a screening before age 50 (i.e., screening could be done at ages 40-44)
[b] Includes individuals aged 45-49 with average risk of colorectal cancer who ever had a screening before age 50 when they were at average risk (i.e., screening could be done at ages 40-44). If a beneficiary was at average risk first then became high risk later, then screening conducted when the individual was at average risk was counted.
[c] Includes individuals aged 45-49 with high risk ever had a screening before age 50 no matter the screening was conducted when he/she was average or high risk.

© 2024 Siddique S et al. *JAMA Network Open*.

eTable 5. Comparing CRC Screening Uptake by Social Deprivation Index within Each Race and Ethnicity Group Among Blue Cross Blue Shield Beneficiaries Aged 45-49 Years During the Post-Recommendation Period (May 2021 to December 2022)

	Quintile of Social Deprivation Index				
	1 (highest SES) [b]	2	3	4	5 (lowest SES)
**Non-Hispanic White**					
Bimonthly Mean Frequency (SD) of Beneficiaries Enrolled	198,144 (7,191)	161,443 (4,663)	146,285 (4,178)	123,808 (3,099)	81,421 (1,318)
Bimonthly Mean Uptake (SD) of CRC Screening	1.82 (0.72)	1.54 (0.63)	1.38 (0.56)	1.28 (0.49)	1.21 (0.42)
P-value comparing with 1st quintile [a]	Ref	0.99	0.19	0.04	0.01
**Non-Hispanic Black**					
Bimonthly Mean Frequency (SD) of Beneficiaries Enrolled	10,442 (253)	8,382 (144)	7,550 (116)	6,417 (94)	4,254 (83)
Bimonthly Mean Uptake (SD) of CRC Screening	1.86 (0.77)	1.51 (0.62)	1.37 (0.59)	1.28 (0.49)	1.21 (0.45)
P-value comparing with 1st quintile	Ref	0.87	0.16	0.05	0.02
**Non-Hispanic Asian**					
Bimonthly Mean Frequency (SD) of Beneficiaries Enrolled	4,596 (248)	3,683 (164)	3,274 (124)	2,702 (131)	1,715 (89)
Bimonthly Mean Uptake (SD) of CRC Screening	1.92 (0.79)	1.53 (0.70)	1.36 (0.60)	1.31 (0.52)	1.23 (0.50)
P-value comparing with 1st quintile	Ref	0.92	0.14	0.08	0.03
**Non-Hispanic Native American**					
Bimonthly Mean Frequency (SD) of Beneficiaries Enrolled	641 (29)	485 (26)	405 (17)	344 (31)	216 (12)
Bimonthly Mean Uptake (SD) of CRC Screening	1.82 (0.77)	1.61 (0.88)	1.44 (0.72)	1.26 (1.02)	1.33 (0.91)
P-value comparing with 1st quintile	Ref	0.99	0.99	0.91	0.92
**Hispanic**					

© 2024 Siddique S et al. *JAMA Network Open*.

Bimonthly Mean Frequency (SD) of Beneficiaries Enrolled	14,742 (776)	12,069 (548)	10,798 (523)	9,092 (441)	5,831 (200)
Bimonthly Mean Uptake (SD) of CRC Screening	1.80 (0.71)	1.53 (0.63)	1.38 (0.56)	1.29 (0.54)	1.24 (0.49)
P-value comparing with 1st quintile	Ref	0.99	0.44	0.14	0.08
**Unknown**					
Bimonthly Mean Frequency (SD) of Beneficiaries Enrolled	272,032 (12,042)	225,061 (8,746)	200,704 (7,503)	168,993 (5,813)	111,198 (3,165)
Bimonthly Mean Uptake (SD) of CRC Screening	1.84 (0.72)	1.56 (0.62)	1.40 (0.56)	1.29 (0.48)	1.18 (0.41)
P-value comparing with 1st quintile	Ref	0.99	0.17	0.03	0.004

[a] P-values are from two-sided t-test comparing mean CRC screening uptake within each race and ethnicity subgroup based on social deprivation index

[b] SES = Socioeconomic Status

© 2024 Siddique S et al. *JAMA Network Open*.

eFigure: Changes in colorectal cancer screening uptake among Blue Cross Blue Shield Axis beneficiaries aged 45-49 years with average risk of colorectal cancer, stratified by screening modalities.

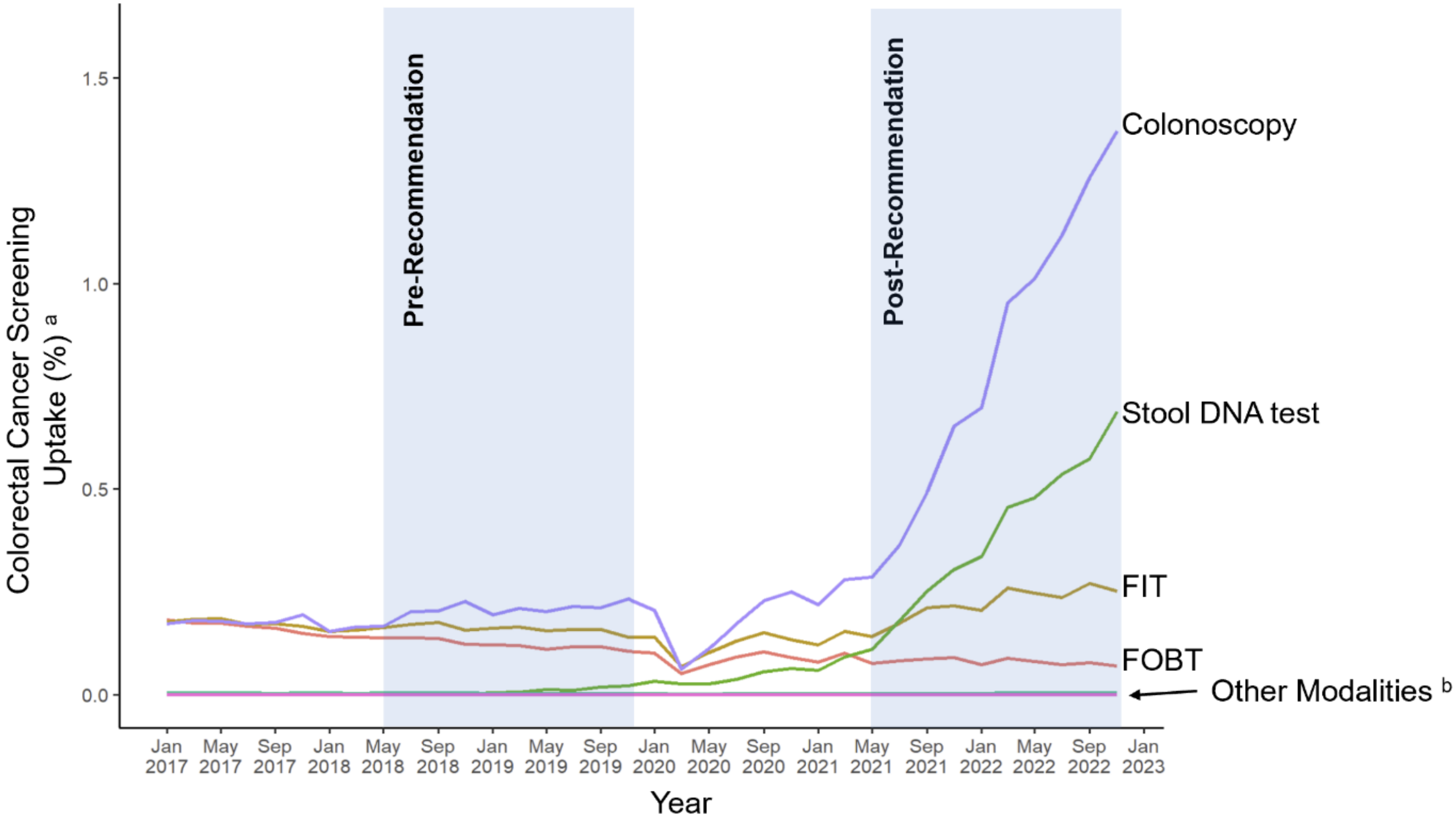

Footnote: [a] Screening uptake was calculated by dividing the number of BCBS beneficiaries aged 45-49 years who had 1) BCBS as the primary insurance for at least 12 months prior to the start of the period and remained enrolled through the end of the 2-month

© 2024 Siddique S et al. *JAMA Network Open*.

period; and 2) did not receive CRC screening or related procedures in the 12 months preceding the 2-month period, by the subset of the denominator who received CRC screening. Footnote: [a] Screening uptake was calculated by dividing the number of BCBS beneficiaries aged 45-49 years who had 1) BCBS as the primary insurance for at least 12 months prior to the start of the period and remained enrolled through the end of the 2-month period; and 2) did not receive CRC screening or related procedures in the 12 months preceding the 2-month period, by the subset of the denominator who received CRC screening. Analyses examining specific screening modalities counted different modalities separately (colonoscopy yes/no, stool DNA test yes/no).

[b] Other modalities include flexible sigmoidoscopy, double-contrast barium enema, and CT colonography.

Abbreviations: FIT: Fecal immunochemical test, FOBT: Fecal occult blood test.

© 2024 Siddique S et al. *JAMA Network Open*.

eReferences

1. Fedewa SA, Star J, Bandi P, et al. Changes in Cancer Screening in the US During the COVID-19 Pandemic. *JAMA Netw Open.* 2022;5(6):e2215490.
2. Mazidimoradi A, Tiznobaik A, Salehiniya H. Impact of the COVID-19 Pandemic on Colorectal Cancer Screening: a Systematic Review. *J Gastrointest Cancer.* 2022;53(3):730-744.
3. El-Shabasy RM, Nayel MA, Taher MM, Abdelmonem R, Shoueir KR, Kenawy ER. Three waves changes, new variant strains, and vaccination effect against COVID-19 pandemic. *Int J Biol Macromol.* 2022;204:161-168.
4. Internal Revenue Service DotT, Employee Benefits Security Administration DoL, Centers for M, Medicaid Services DoH, Human S. Coverage of certain preventive services under the Affordable Care Act. Final rules. *Fed Regist.* 2013;78(127):39869-39899.
5. Engel-Nitz NM, Miller-Wilson LA, Le L, Limburg P, Fisher DA. Colorectal screening among average risk individuals in the United States, 2015-2018. *Prev Med Rep.* 2023;31:102082.
6. Rex DK, Boland CR, Dominitz JA, et al. Colorectal Cancer Screening: Recommendations for Physicians and Patients from the U.S. Multi-Society Task Force on Colorectal Cancer. *Am J Gastroenterol.* 2017;112(7):1016-1030.
7. Shaukat A, Kahi CJ, Burke CA, Rabeneck L, Sauer BG, Rex DK. ACG Clinical Guidelines: Colorectal Cancer Screening 2021. *Am J Gastroenterol.* 2021;116(3):458-479.

© 2024 Siddique S et al. *JAMA Network Open*.
