# Supplementary material for: USPSTF Colorectal Cancer Screening Recommendation and Uptake for Individuals Aged 45 to 49 Years
Source: JAMA Netw Open. 2024 Oct 3;7(10):e2436358. doi: 10.1001/jamanetworkopen.2024.36358 (PMC11450516; doi:10.1001/jamanetworkopen.2024.36358)
Supplement: Supplement 2. — Data Sharing Statement [file jamanetwopen-e2436358-s002.pdf]

Data Sharing Statement

Siddique. USPSTF Colorectal Cancer Screening Recommendation and Uptake for Individuals Aged 45 to 49 Years. *JAMA Netw Open*. Published October 03, 2024. doi:10.1001/jamanetworkopen.2024.36358

Data
Data available: No

Additional Information
Explanation for why data not available: We obtained access to the BCBS Axis data through an institutional agreement on research collaboration between Yale University and BCBS and a proposal describing the work presented in this manuscript that was approved by the BCBS. We are not allowed to share the raw data as stipulated by the institutional agreement and the proposal. Interested investigators should contact BCBS directly for potential data access. On the other hand, we are open to sharing our analytical methods and statistical programs with any researchers who contact us.
